# Characteristics of Clinics without National Health Insurance Contracts: A Nationwide Survey in Taiwan

**DOI:** 10.3390/ijerph19031517

**Published:** 2022-01-28

**Authors:** Pei-Jyun Lu, Jui-Yao Liu, Hsin Ma, Tzeng-Ji Chen, Li-Fang Chou, Shinn-Jang Hwang

**Affiliations:** 1 Department of Medical Education, Taipei Veterans General Hospital, Taipei 112, Taiwan; lupeggy8605@gmail.com; 2 Department of Family Medicine, National Yang Ming Chiao Tung University Hospital, Yilan 260, Taiwan; liujuiyao@gmail.com; 3 School of Medicine, National Yang Ming Chiao Tung University, Taipei 112, Taiwan; sjhwang@vghtpe.gov.tw; 4 Department of Family Medicine, Taipei Veterans General Hospital, Taipei 112, Taiwan; tov8588@gmail.com; 5 Institute of Hospital and Health Care Administration, School of Medicine, National Yang Ming Chiao Tung University, Taipei 112, Taiwan; 6 Big Data Center, Department of Medical Research, Taipei Veterans General Hospital, Taipei 112, Taiwan; 7 Department of Public Finance, National Chengchi University, Taipei 116, Taiwan; lifang@nccu.edu.tw

**Keywords:** ambulatory care facilities, national health programs, private practice, Taiwan

## Abstract

Based on the 1978 Alma-Ata Declaration, the key to achieving health for all is primary health care, and many countries have established various comprehensive health care systems. Because of the financial toll of a public health care system, government-sponsored public health insurance is not universally accepted. This study used Taiwan as the backdrop to understand why many health clinics have chosen not to accept the National Health Insurance (NHI), despite it covering 99.93% of the country’s population. The clinics’ operational details were garnered from the datasets of Taiwan’s open government data platforms and checked against the list of contracting clinics within the NHI. Of 10,907 Western medicine primary care clinics in 2016, as many as 9846 (90.3%) clinics had signed contracts with the NHI. The remaining 1061 noncontracting clinics were distributed in urban (94.5%, *n* = 1003), suburban (4.9%, *n* = 52), and rural/remote areas (0.6%, *n* = 6). The NHI did not have contracts with 183 plastic surgery, 88 internal medicine, and 85 surgery clinics. In conclusion, nearly one-tenth of clinics practiced independently of the NHI in Taiwan. Their reasons for declining the contract and practices for delivering their services deserve further studies.

## 1. Introduction

The International Conference on Primary Health Care, known as the Declaration of Alma-Ata, expressed the need to protect and promote health for all people worldwide. It declared that health is a socioeconomic issue and is a basic human right [1]. Since the conference, many international governments have applied different types of health care systems in their countries to achieve this goal. Countries such as the United Kingdom, Australia, Denmark, Finland, Greece, Iceland, Ireland, Italy, Latvia, New Zealand, Norway, Portugal, Spain, and Sweden have implemented the National Health Service (NHS), which offers widespread coverage and is financed by general tax revenue. NHS has a high coverage rate, mostly 100%, and the physician payment system is based on a salary and per capita payment system. The relatively low number of acute care beds indicates a high degree of control over the supply of health care services, resulting in better cost control and a lower ratio of health care spending to GDP. The public’s access rate, hospitalization rate, and average length of stay are lower than those of NHI countries, but the public has higher waiting periods, less choice of care, less efficient institutions, and lower public satisfaction. Countries such as Austria, Belgium, Canada, Colombia, Costa Rica, Czech Republic, Estonia, France, Germany, Hungary, Israel, Japan, Lithuania, Luxembourg, Netherlands, Poland, Turkey, Slovakia, Slovenia, and South Korea use National Health Insurance (NHI), mandatory comprehensive insurance financed by insurance premiums. NHI countries have a high level of public satisfaction, but face the dilemma of oversupply and overconsumption and high medical costs. In addition, there are different insurance systems in the same country, with different payments and benefits, and some groups are uninsured, leading to disputes over fairness [2,3,4].

Whether by instituting the NHS, the NHI, or other health care systems, countries have organized their individual health care systems differently and, accordingly, each country’s insurance coverage, funding sources, health care provisions, and regulatory systems vary [5]. In most countries, health care providers are either employed or are contracted by the state. As the vast majority of health care costs are expensive, public health care does not cover all medical conditions. Most public medical services provide coverage for the most basic medical expenses, with certain limits and auditing standards. In such a limited and restricted environment, there is adequate need and demand by individuals who can pay for private health care [6,7].

This study aimed to use Taiwan as an example to understand how many clinics in NHI-established countries choose not to enroll in public health insurance by using detailed statistics and analyses of geographical distributions and specialties. Many studies have been conducted to determine how many people pay for private health insurance, how much people pay for private health insurance, and the reasons why people seek private health insurance [8,9]. Statistics about self-paid medical practices are not widely available, and most of the relevant research to date is accomplished through questionnaires [10,11]. The results of this study can be used as a basis for medical service systems and international comparisons, and may facilitate the formulation of future health policies.

## 2. Materials and Methods

### 2.1. Background

Taiwan’s NHI program was established in 1995, and for 26 years the Ministry of Health and Welfare has served as its competent authority. Before 1995, there were different social health insurance schemes covering specific groups of people, including Labor Insurance, Government Employees Insurance, Farmer’s Insurance, Low-Income Household Insurance, and so on. The coverage was 59 percent at that time. The majority of those not covered are children younger than fourteen and seniors older than sixty-five. However, these groups have the greatest medical needs [12,13]. The NHI system aims to protect the health of the entire population through a self-help, mutual assistance system. The goal is to provide equal access to health care resources for all residents and to control the overall cost of health care [14]. In addition to serving people within the Republic of China (ROC), after many amendments to the law, the NHI has gradually included non-nationals who have been registered in Taiwan for six months to be eligible for public health insurance. As of the end of June 2020, the total number of people covered by the NHI was 23,954,568. The insurance premiums are shared by the insured and the government. The medical services provided include outpatient care, hospitalizations, Chinese medicine, dentistry, services related to childbirth, rehabilitation, home care, and chronic mental illness rehabilitation. The scope of coverage includes diagnosis and treatment, examination, inspection, consultation, surgery, anesthesia, medicine, supplies, treatment, nursing, and hospital stays, including all necessary diagnosis and treatment services [13,14,15].

The health care system in Taiwan includes both public and private hospitals. According to statistics from the Ministry of Health and Welfare in 2020 [16], there were a total of 479 public and private hospitals, and the average daily total number of outpatient visits was 406,575. Hospitals have an outpatient and emergency system, and inpatients are referred from the hospitals’ outpatient departments or from other affiliated clinics. There is no mandatory family medicine system, and patients are free to choose their own medical specialty. On 9 June 2010, the regulations for the division and examination of specialist physicians were amended and published. Article 3 of the regulations was revised to include family medicine, internal medicine, surgery, pediatrics, obstetrics and gynecology, orthopedics, neurosurgery, urology, otorhinolaryngology, ophthalmology, dermatology, neurology, psychiatry, rehabilitation, anesthesiology, diagnostic radiology, radiation oncology, anatomical pathology, clinical pathology, nuclear medicine, emergency medicine, occupational medicine, and plastic surgery as medical specialties, equaling a total of 23 specialties [17]. About 63% of medical staff are employed in public hospitals and receive a fixed salary. The remaining medical staff serve in private hospitals [18]. In accordance with the provisions of the NHI Medical Service Organization Special Agreement and Management Method under the NHI Law, all licensed medical institutions can apply to the government to join the NHI Contract Clinic. The contract is valid for three years and can be renewed after the expiration date if there are no violations [19]. Contracted medical service institutions include contracted hospitals, clinics, pharmacies, and other medical service institutions (medical examination laboratories, home care institutions, midwifery institutions, psychiatric rehabilitation institutions, physiotherapy laboratories, occupational therapy laboratories, medical radiology laboratories, and respiratory care laboratories) designated by the competent authorities [14].

According to the statistics of the Civil Affairs Bureau of the government of Taiwan’s capital, Taipei City, the city’s total population was 2,544,720 in September 2021. Taipei City is also the center of northern Taiwan, and its surrounding areas include New Taipei City, Keelung, and Taoyuan, with a combined population of more than one-third of Taiwan’s total population. According to the Taiwan Medical Association, in 2020, there were 1772 medical institutions in Taipei, which is the most in Taiwan, including 10 medical centers [20].

However, despite the widespread number of medical institutions in Taiwan and the seemingly accessible nature of government-subsidized public health care, private medical facilities are prevalent. Some of these private health care facilities are not even part of the NHI system. Reasons for individuals’ preferences for private health insurance stem from personal preferences, such as the desire for a better quality of care and the refusal to accept long wait times. Furthermore, medical staff and institutions are motivated to contract with private insurance, as higher incomes are possible for employees and better medical staff/doctor–patient relationships can be fostered in this environment [21,22].

### 2.2. Data Sources

Data were collected on 2 September 2016. Taiwan’s open government data platform was the main source of analytical data for this study. We also refer to the websites of the NHI Administration of the Ministry of Health and Welfare, the Ministry of the Interior of the ROC, and the study of urbanization stratification of rural and urban areas in Taiwan for research analysis and comparison. The relevant data are described as follows:

#### 2.2.1. The Open Government Data Platform

Taiwan’s open government data platform is an interdepartmental project established by the ROC’s government in accordance with the provisions of the Government Information Disclosure Act and promoted by the Executive Yuan since 2013. Anyone (including enterprises) is free to use the open data provided by the platform within the limits of their use. The dataset contains 18 categories of information: reproductive health, birth and adoption, schooling and education, military service, job search and employment, starting a career, marriage, investment and finance, leisure travel, transportation and communication, medical care, home purchases and migration, election and voting, safety and quality of life, retirement, senior care, life rituals, and public information [23].

#### 2.2.2. The NHI Administration Ministry of Health and Welfare Website

We used the “Compressed List of Health Insurance Contracted Medical Institutions’’ in the contracted medical services on the NHI Administration Ministry of Health and Welfare website. It contains the branch office, medical institution code, medical institution name, institution address, telephone area code, telephone number, special contract category, type of medical institution, date of termination or discontinuation of business, and “Compressed File of Service Details of Medical Institutions” and “Compressed File of Practice Division of Medical Institutions” (three files altogether). The comparison files of each code were analyzed, including five files named “Division business group code comparison file”, “Special category code comparison file”, “Type code comparison file”, “Service item code comparison file”, and “Diagnosis and treatment department code comparison file” [24].

#### 2.2.3. Ministry of the Interior Website

The monthly statistical report of the Ministry of the Interior on the website of the Ministry of the Interior of the ROC contains a collection of data categorized into eight items: household affairs, civil affairs, cooperative undertakings and people’s organizations, land affairs, police affairs, immigration, firefighting, and construction [25].

#### 2.2.4. Incorporating Development Stratification of Taiwan Townships into Sampling Design of Large-Scale Health Interview Survey

Liu et al. studied the stratification of urbanization in towns and cities in Taiwan. The 359 rural–urban areas in Taiwan were divided into 7 clusters based on population density, number of people with a post-secondary degree or higher, number of people aged 65 or older, agricultural population, and number of Western medicine practitioners per 100,000 people. In the study, the order of the clusters from first to seventh were highly urbanized towns, moderately urbanized towns, emerging towns, general towns and cities, aging towns, agricultural towns, and remote towns [26].

### 2.3. Data Processing

This study first used basic information from medical institutions and medical personnel in the open government data platform (Section 2.2.1), updated about every six months by the Department of Medical Services of the Ministry of Health and Welfare. The study was based on government-related statistics (files in .csv format) retrieved from the platform on 2 September 2016 [23]. The data included 22,219 items, including Western medicine clinics and Chinese medicine, dentistry, health clinics and hospitals. The information included the name, ownership, type, county, city, town, telephone number, address, department, and the number of the medical staff of the clinic. Of these, 10,907 Western medical clinics were selected, excluding Chinese medicine, dentistry, and health clinics and hospitals. Next, we used the website of the NHI Administration Ministry of Health and Welfare (Section 2.2.2) to search for “health insurance contracted medical institutions” and to determine which clinics were not enrolled in the NHI. According to the clinical department corresponding to each “code comparison file” used to query and reanalyze the classification, the relevant clinics’ initial classification was divided into 23 specialties. The clinics not enrolled in the NHI were classified according to the “rural–urban population and statistics by metropolitan area” in the category of “household administration” on the Ministry of Interior of the ROC (Section 2.2.3), which is the closest to this study’s data period. Last, the urbanization level of the clinics’ locations was categorized according to the ‘’Incorporating Development Stratification of Taiwan Townships into Sampling Design of Large-Scale Health Interview Survey’’ (Section 2.2.4).

### 2.4. Statistical Analysis

We manually input our collected data and performed our analysis with Microsoft Excel 365 (Microsoft Inc., Redmond, WA, USA). The descriptive statistics show the results.

### 2.5. Ethical Approval

The data obtained for this study are all public. Under the Personal Data Protection Act and human research regulations in Taiwan, this study does not require Institutional Review Board review.

## 3. Results

**Figure 1:**
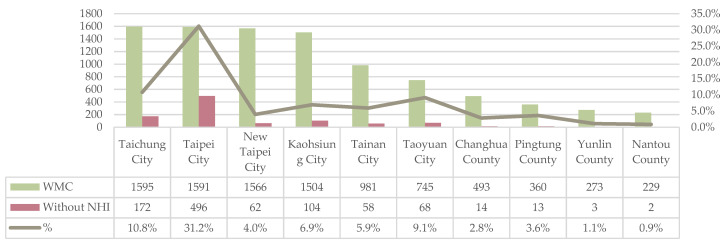
Geographical distribution of clinics without health insurance. WMC: Western Medical Clinic.

Among the 10,907 Western medicine clinics, 1061 clinics (9.7%) did not participate in the NHI. According to the geographical distribution analysis, clinics not enrolled in the NHI ranged from 0.9% to 31.5%, with an average proportion of 7.6% and standard deviation of 0.089. A total of 496 clinics not enrolled in the NHI were located in Taipei City, accounting for 31.2% of all the Western clinics in the area, followed by Taichung City with 172 clinics for 10.8% of all clinics in the area. There were fewer than 100 other municipalities (New Taipei City, Kaohsiung City, Tainan City, and Taoyuan City), accounting for between 5% and 9% of Western medical clinics in the region. Other counties and cities accounted for less than 5% of Western medical clinics (Figure 1) (Table S1).

**Figure 2:**
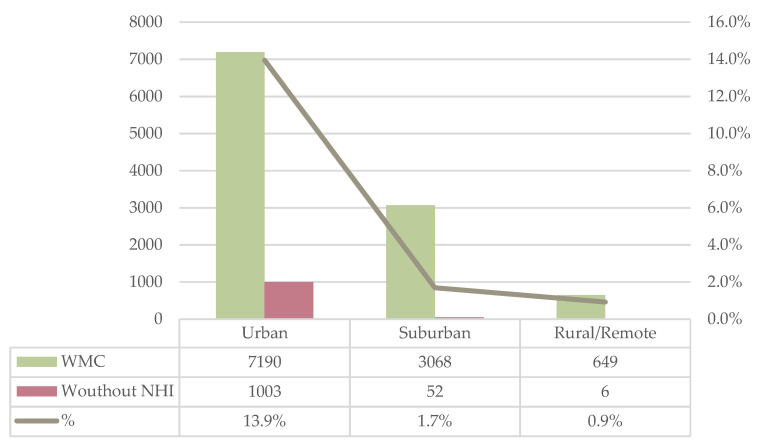
Urbanization level of the location of clinics without health insurance. WMC: Western Medical Clinic.

According to the analysis of its degree of urbanization, noncontracting clinics were distributed in urban (94.5%, *n* = 1003), suburban (4.9%, *n* = 52), and rural/remote areas (0.6%, *n* = 6). The noncontracting ratio was highest in urban areas with 13.9%, followed by suburban (1.7%) and rural/remote (<1%) areas (Figure 2) (Table S2). The mean value was 5.5%.

**Figure 3:**
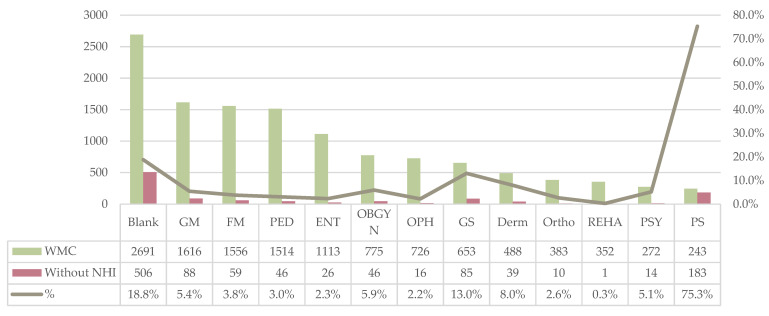
Medical specialties of clinics without health insurance. WMC: Western medical clinic; GM: general medicine; FM: family medicine; PED: pediatrics; ENT: otorhinolaryngology; OBGYN: obstetrics and gynecology; OPH: ophthalmology; GS: surgery; Derm: dermatology; Ortho: orthopedics; REHA: rehabilitation; PSY: psychiatry; PS: plastic surgery.

As to specialties, clinics not enrolled in the NHI ranged from 0.3% to 75.3%, with an average proportional of 11.2% and standard deviation of 0.199. The NHI did not have contracts with 183 plastic surgery clinics, followed by 88 internal medicine, 85 surgery, and 59 family medicine clinics. The noncontracting ratio of the 243 practicing plastic surgery clinics reached a disproportionate value of 75.3%. There were also many unspecialized medical professionals, accounting for 18.8% of the medical professionals. The rest were mostly below 10%. The data revealed that no clinics were accepting private insurance in radiology, nuclear medicine, occupational medicine, anatomical pathology, and clinical pathology (Figure 3) (Table S3).

## 4. Discussion

According to the data analysis, Taipei City (31.8%) had the most clinics accepting only private insurance in the region. Taipei City is the primary development center in Taiwan’s financial, political, economic, cultural, and educational fields. In addition to Taipei City, we found that in the other five municipalities in Taiwan—New Taipei City, Taoyuan City, Taichung City, Tainan City, and Kaohsiung city— the proportion of clinics without NHI contracts was also high.

In the urbanization analysis, we found that urban areas with high population densities, high levels of education, and high densities of medical resources also had the highest proportion of clinics accepting private insurance. The higher economic capacity, higher spending power, and greater receptiveness to new information in this cluster may explain the higher prevalence of clinics accepting private insurance. There are 70 urban towns in these urban areas, and Taipei City has the largest number of cities and counties in the distribution, consistent with the results seen in Figure 1.

In terms of specialty clinics and medical facilities observed from the data, plastic surgery was the major specialty (75.3%). The 21st century is the era of imaging. The development of mobile phones and cameras allows us to pick up images at any time. Therefore, people in any industry attach importance to appearance packaging, making the medical aesthetics industry flourish. Plastic surgery ranges from eyelid surgery and breast augmentation to laser surgery for varicose veins. In addition to plastic surgery, integrative medicine, preventive medicine, weight loss, hair transplantation, and hair growth are also emerging. In addition, a small number of clinics only accepting private insurance have specialized even further into focusing on specific procedures, such as high-level ultrasounds, or operating in specific niches, such as psychotherapy or self-paid health checkups. We also found that unspecialized clinics accounted for about 50% of all clinics without NHI contracts, as cosmetic services are not included in the 23 NHI-covered specialties. These data show that clinics unrestricted by NHI contracts covering only certain specialties can be more diversified in the procedures and services that they offer. Therefore, the total number of clinics with specialty counted is higher than the total number of self-paid clinics.

As this study is a quantitative analysis, we did not conduct an in-depth study on why people chose to seek self-paid medical services. Previous research has identified a possible reason as being people’s dissatisfaction with public health services. Because of the large number of patients in NHI hospitals and clinics, patients experience long wait times. Furthermore, people have also expressed sentiments that the service in public hospitals is worse than that in private medical clinics [22]. Because the doctor-to-patient ratio is small in private clinics, doctors have ample time to devote to their patients, assess their conditions, and discuss treatments. Nonemergency surgeries can be booked in advance in private clinics, thus making operations more efficient [21]. In addition to the long wait times at NHI hospitals, inpatient beds are hard to find. A study in the United Kingdom (UK) pointed out that more and more older patients have short-term hospitalization needs, and most in this age group have sufficient financial means. Self-paid care could meet their need for short-term hospitalizations [27]. Presently, people often have the freedom to choose their medical care. Most people are more financially capable of paying for their medical care without being limited by the cost of the visit [28]. Studies have also shown that a higher proportion of affluent people choose self-paid health care over public health care services [29]. Under the NHI system, after implementing a total budget limit in July 2002, health insurance does not currently cover all medications and tests. Thus, some of the more expensive medications and tests are not offered in NHI-contracted hospitals, so people still need to go to self-paid clinics for help [30].

Moreover, medical professionals and staff tend to choose employment in private hospitals and clinics because of salary limitations. Since the NHI was launched in 2005, the ratio of health care spending to the GDP has decreased each year. The government sector’s health care spending decreased from 6.3% of the GDP in 1995 to 4.3% of the GDP in 2016. Although the low cost of premiums has made health care cheaper, it has also led to a waste of health care resources and sweatshops. Physicians prefer to spend more time on self-paid medical procedures because they can generate more revenue than they could if they performed similar procedures in NHI-contracted facilities [21]. In addition, because of the lower number of patients needing to be seen in private clinics, the physical caseload for physicians is dramatically less than it would be in NHI-contracted facilities [30]. As many individuals/patients have noted, physicians have echoed that the fewer patients they have, the more time they have to establish better patient rapports, and thereby gain a higher sense of accomplishment with individual cases [31].

In self-financed medical care, some clinics accept health insurance and offer self-paid care simultaneously, which is called “dual practice”. In our statistics, these clinics are classified as health insurance clinics, so the number of clinics that perform self-paid services is underestimated. Previous studies have suggested the implementation of dual practice has resulted in a decline in the overall quality of care. However, this conclusion is set in the context of a specific market, such as in a predominately wealthy area. Affluent people prefer to spend money on private, self-paid care instead of public health services [32]. In general, there is no clear evidence that dual practice negatively affects public health services [5,33].

Furthermore, it is important to consider whether the increasing number of self-paid health clinics affects the NHI’s operation. Studies in Norway have shown that the increasing number of people with private health insurance does not affect the public’s support of public health services, and there is no correlation between the two [34]. However, there may be some impact on supply and demand, as the public freely chooses the supply and demand of health care services [35]. In terms of average life expectancy and quality of life, the low premiums of health insurance have increased the accessibility of medical care, allowing many people with lower financial means to have access to medical services, which in turn has increased the average life expectancy of the population [36]. In contrast, the goal of self-paid health care focuses on improving patients’ quality of life rather than on prolonging their lives [35]. However, this does not mean that self-paid care is less effective than the NHI in treating patients, as studies have shown no significant difference in the quality of procedures performed in either self-paid clinics or NHI-contracted facilities [37]. Patient satisfaction with postoperative care was higher in self-paid clinics than in NHI-contracted facilities [38]. In addition, patients in private clinics may have a higher chance of being operated on by a senior attending physician. However, for more difficult and complex cases in NHI hospitals, a senior attending physician will also operate [39]. Self-paid clinics also have some positive benefits for the NHI. For example, a study in the UK noted that more than 40% of long-term inpatients were placed in freestanding facilities, which relieved the NHS of a significant burden in terms of long-term care for the elderly, chronically mentally ill, and mentally disabled [21].

### Limitations

From the open government data platform, we were limited by not knowing the included physicians’ age, gender, or family status, or the number of years of their employment. Some of the uninsured clinics included in the data are currently out of business, but have not been removed from the data. The latest data of basic information from medical institutions and medical personnel in Taiwan’s open government data platform pertained to 2020. Considering the effects of the coronavirus disease beginning in 2019, we chose the older but stable data of 2016. As a cross-sectional analysis, this study did not include some of the newly opened clinics that have not yet obtained health insurance contracts, which may cause statistical errors. In addition, we do not know the actual practice content and performance of the clinics. Because this study was a quantitative analysis, we could not understand why physicians and facilities chose not to sign up for the NHI. There was also no analysis of the age, sex, or socioeconomic status of the people who visited self-paid clinics, or of why they chose to visit them. The degree of urbanization used in our analysis is taken from a 2000 study, which may not reflect the current urban and rural changes.

## 5. Conclusions

The descriptive analysis of this study found that the distribution of self-paid clinics was highest in urban areas with high population densities, residents with higher education degrees, and high medical-resource density. Among them, the capital city of Taiwan, Taipei City (31.8%), had the most self-paid clinics. In terms of self-paid or private specialty clinics, plastic surgery was the most common (75.3%) private specialty clinic. General medical clinics accounted for about 50% of all self-paid clinics; however, as some of the clinics documented in the data were dual-practice clinics accepting both NHI and private insurance, the total number of self-paid clinics was underestimated. Since this study is a quantitative analysis, we cannot know the reasons why physicians choose not to sign up for health insurance. These will be investigated in a more detailed study.

## Data Availability

Data are contained within the article and Supplementary Materials.

## Supplementary Materials

The following supporting information can be downloaded at: https://www.mdpi.com/article/10.3390/ijerph19031517/s1, Table S1: Geographical distribution of clinics without health insurance of 21 counties in Taiwan; Table S2: Urbanization level of the location of clinics without health insurance; Table S3: Medical specialties of clinics without health insurance.
