# Supplementary material for: Characteristics of Clinics without National Health Insurance Contracts: A Nationwide Survey in Taiwan
Source: Int J Environ Res Public Health. 2022 Jan 28;19(3):1517. doi: 10.3390/ijerph19031517 (PMC8835185; doi:10.3390/ijerph19031517)
Supplement: Supplementary file 1 [file ijerph-19-01517-s001.zip › ijerph-1519874-supplementary.pdf]

**Table S1.** Geographical distribution of clinics without health insurance of 21 counties in Taiwan

| County | WMC | Without NHI | % |
|---|---|---|---|
| Taichung City | 1595 | 172 | 10.8% |
| Taipei City | 1591 | 496 | 31.2% |
| New Taipei City | 1566 | 62 | 4.0% |
| Kaohsiung City | 1504 | 104 | 6.9% |
| Tainan City | 981 | 58 | 5.9% |
| Taoyuan City | 745 | 68 | 9.1% |
| Changhua County | 493 | 14 | 2.8% |
| Pingtung County | 360 | 13 | 3.6% |
| Yunlin County | 273 | 3 | 1.1% |
| Nantou County | 229 | 2 | 0.9% |
| Hsinchu City | 209 | 22 | 10.5% |
| Chiayi City | 209 | 15 | 7.2% |
| Hsinchu County | 190 | 16 | 8.4% |
| Miaoli County | 187 | 1 | 0.5% |
| Yilan County | 171 | 3 | 1.8% |
| Chiayi County | 154 | 0 | 0.0% |
| Keelung City | 152 | 5 | 3.3% |
| Hualien County | 139 | 3 | 2.2% |
| Taitung County | 80 | 2 | 2.5% |
| Penghu County | 50 | 0 | 0.0% |
| Kinmen County | 29 | 2 | 6.9% |
| **Total** | **10907** | **1061** | **9.7%** |

WMC: Western medical clinic

**Table S2.** Urbanization level of the location of clinics without health insurance

| Urbanization Level | WMC | Without NHI | % |
|---|---|---|---|
| Cluster 1 | 3399 | 666 | 19.6% |
| Cluster 2 | 3791 | 337 | 8.9% |
| Cluster 3 | 1695 | 34 | 2.0% |
| Cluster 4 | 1373 | 18 | 1.3% |
| Cluster 5 | 99 | 0 | 0.0% |
| Cluster 6 | 245 | 2 | 0.8% |
| Cluster 7 | 305 | 4 | 1.3% |
| **Total** | **10907** | **1061** | **9.7%** |

WMC: Western medical clinic

**Table S3.** Medical specialties of clinics without health insurance

| Specialties | WMC | Without NHI | % |
|---|---|---|---|
| Blank | 2691 | 506 | 18.8% |
| GM | 1616 | 88 | 5.4% |
| FM | 1556 | 59 | 3.8% |
| PED | 1514 | 46 | 3.0% |
| ENT | 1113 | 26 | 2.3% |
| OBGYN | 775 | 46 | 5.9% |
| OPH | 726 | 16 | 2.2% |
| GS | 653 | 85 | 13.0% |
| Derm | 488 | 39 | 8.0% |
| Ortho | 383 | 10 | 2.6% |
| REHA | 352 | 1 | 0.3% |
| PSY | 272 | 14 | 5.1% |
| PS | 243 | 183 | 75.3% |
| Neuro | 125 | 11 | 8.8% |
| GU | 67 | 7 | 10.4% |
| NS | 18 | 3 | 16.7% |
| ANES | 7 | 4 | 57.1% |
| ER | 6 | 3 | 50.0% |
| RAD | 2 | 0 | 0.0% |
| NM | 1 | 0 | 0.0% |
| Occup | 1 | 0 | 0.0% |
| AP | 1 | 0 | 0.0% |
| CP | 1 | 0 | 0.0% |
| **Total** | **12611** | **1147** | **9.1%** |

WMC: Western medical clinic; GM: general medicine; FM: family medicine; PED: pediatrics; ENT: otorhinolaryngology; OBGYN: obstetrics and gynecology; OPH: ophthalmology; GS: surgery; Derm: dermatology; Ortho: orthopedics; REHA: rehabilitation; PSY: psychiatry; PS: plastic surgery; Neuro: neurology; GU: urology; NS: neurosurgery; ANES: anesthesiology; ER: emergency medicine; RAD: radiology; NM: nuclear medicine; Occup: occupational medicine; AP: anatomical pathology; CP: clinical pathology
